# Characterization of Carbonic Anhydrase In Vivo Using Magnetic Resonance Spectroscopy

**DOI:** 10.3390/ijms21072442

**Published:** 2020-04-01

**Authors:** Jyoti Singh Tomar, Jun Shen

**Affiliations:** Molecular Imaging Branch, National Institute of Mental Health, NIH, Bethesda, MD 20892, USA

**Keywords:** in vivo MRS, carbonic anhydrase, GABAergic transmission, neurological diseases, psychiatric diseases

## Abstract

Carbonic anhydrase is a ubiquitous metalloenzyme that catalyzes the reversible interconversion of CO_2_/HCO_3_^−^. Equilibrium of these species is maintained by the action of carbonic anhydrase. Recent advances in magnetic resonance spectroscopy have allowed, for the first time, in vivo characterization of carbonic anhydrase in the human brain. In this article, we review the theories and techniques of in vivo ^13^C magnetization (saturation) transfer magnetic resonance spectroscopy as they are applied to measuring the rate of exchange between CO_2_ and HCO_3_^−^ catalyzed by carbonic anhydrase. Inhibitors of carbonic anhydrase have a wide range of therapeutic applications. Role of carbonic anhydrases and their inhibitors in many diseases are also reviewed to illustrate future applications of in vivo carbonic anhydrase assessment by magnetic resonance spectroscopy.

## 1. Introduction

Carbonic anhydrase (CA, also known as carbonate dehydratase or carbonic dehydratase) is a family of enzymes that are present in many different isoforms or carbonic anhydrase-related proteins. CO_2_ is a toxic by-product of cellular respiration, so it needs to be removed from the body. CA catalyzes the interconversion between carbon dioxide and bicarbonate anion, a reaction that occurs very slowly in the absence of CA:

carbon dioxide + H_2_O ↔ H^+^ + bicarbonate

The catalytic action by CA permits near equilibrium between CO_2_ and bicarbonate [1]. The change catalyzed by CA is an interconversion between the nonpolar gaseous carbon dioxide and the conjugate base of carbonic acid, the bicarbonate ion. The exchange between CO_2_ and bicarbonate is almost instantaneous in the presence of CA. In mammals, carbon dioxide gas generated by cellular metabolism leaves the body by the action of red blood cells which rapidly convert it to bicarbonate ion via CA catalysis for transport. Then the bicarbonate ions are converted back to carbon dioxide to be exhaled. CA is a metalloenzyme that exists ubiquitously in seven families: α, β, γ, δ, ζ, θ, and η [2]. These families differ in their preference for metal ions used for performing catalysis. 

In mammals sixteen different isoforms of α-CA (CA I, II, III, IV, VA, VB, VI, VII, IX, XII, XIII, XIV CA XV and CARP VIII, CARP X, and CARP XI) have been identified. These isoforms differ in catalytic activity, their subcellular localization, tissue distribution and sensitivity toward inhibitors. CA I, II, III, VII, XIII exist in cytoplasm; CA VA, VB in the mitochondria, CA IV, IX, XII, XIV, XV in plasma membrane and CA VI is secreted with saliva [3]. CARPs [4] expression is identified in central nervous system (CNS) but their physiological role in CNS is not well established [5]. CARPs lack classical CA activity due to absence of the histidine residue required for catalysis. CARP VIII is associated with motor coordination. Mutation in CARP VIII gene has been associated with ataxia, mental retardation and quadrupedal gait, motor dysfunction, and altered calcium dynamics [6].

Expression levels of CA have been considered as biomarkers in many clinical studies. Several CAs (CA II, IX, XII, and CARPs VIII and XI) are linked with cancer progression and response to cancer chemotherapy [7,8,9,10,11,12]. For example, expression of CA isoform IX is strongly upregulated in several types of tumors including ependymomas, mesotheliomas, follicular carcinomas [13,14,15,16,17,18,19,20] and brain tumors [21,22]. Abnormalities in CA III have been found to be associated with acute myocardial infarction, post infarction treatment efficacy and perioperative myocardial complications [23,24]. CA II, III, IV, and VII are expressed in nervous tissue [25,26]. Isoform CAV II is linked to cellular ion homeostasis and susceptibility to epileptogenesis [27]. 

Overall, CA activity regulates pH and CO_2_ homeostasis, electrolyte secretion and transport, many biosynthetic reactions (e.g., gluconeogenesis, lipogenesis, and ureagenesis), bone resorption, calcification, and tumorigenicity [28]. In the brain there is a general lack of significant CA activities in neurons. Because neurons are metabolically highly active, neuronal CA would hinder the rapid removal of the freely diffusible carbon dioxide through cell membranes [29,30]. The compartmentation of CA in the brain leads to the hydration of carbon dioxide to bicarbonate predominantly in glial cells. As a result, glial cells act as sinks of carbon dioxide [31]. It has been hypothesized that glial hydration of carbon dioxide and transfer of energy with high neuronal activity are coupled to uptake of glutamate by glia [32]. In the brain, CA has also been found to modulate GABAergic excitation, long-term synaptic transformation, attentional gating of memory storage, and cerebrospinal fluid formation [33,34,35]. 

Historically, assessing carbonic anhydrase activities required biopsied tissues and in vitro techniques, making it impossible to study brain CA function and dysfunction in vivo. In contrast to in vitro techniques, magnetic resonance spectroscopy (MRS) allows non-invasive detection of specific biologically relevant molecules in vivo [36]. It has become a very useful and versatile tool for both clinical and basic science studies because it can measure concentrations of many important endogenous and exogenous molecules [37]. Our laboratory discovered the phenomenon of in vivo enzyme-specific ^13^C magnetization transfer [38,39,40,41,42,43] and developed in vivo ^13^C magnetization transfer MRS techniques for measuring carbonic anhydrase-catalyzed interconversion between carbon dioxide and bicarbonate [40]. We first quantified the in vivo rate of bicarbonate dehydration in the rodent brain and the effect of acetazolamide administration on the catalytic action of CA [40]. Recently we have succeeded in measuring brain CA in healthy human subjects [43].

In this article we review in vivo MRS theories and techniques for detecting carbonic anhydrase activities. The implications of CA in neurological and psychiatric disorders and clinically applicable carbonic anhydrase inhibitors (such as acetazolamide) will be discussed in the context of future clinical applications of in vivo MRS characterization of carbonic anhydrase. These will include clinical application of CA inhibitors (CAIs) in brain disorders such as schizophrenia and bipolar disorder [44,45,46,47,48,49,50,51,52,53,54,55,56,57]. As abnormalities in CA are widespread and many drugs target or act on CA, noninvasive in vivo MRS techniques are poised to play an important role in characterizing and elucidating the function and dysfunction of carbonic anhydrase in many brain disorders as well as in monitoring treatment. 

## 2. In Vivo Magnetic Resonance Spectroscopy (MRS) for Studying Carbonic Anhydrase

CA expression level is an important biomarker and its association with several diseases is well established [58]. Many CA isoforms are either upregulated or downregulated under pathological conditions. As CA function depends on microenvironments (tumor cell, nerve cell, blood cell, lungs cell), estimation of enzyme expression from excised tissue may not accurately reflect abnormalities of its catalytic functions. Therefore, techniques that can measure in vivo CA activities are highly desirable. In vivo MRS can measure the rate of enzyme-catalyzed reactions using magnetization (or saturation) transfer method. When kinetically relevant reporter molecules are spin labeled with repetitive saturation of their exchange partner molecules to gain enough SNR, the exchange rate can be quantified from signal change and longitudinal relaxation time (*T*_1_) of the reporter molecules. By introducing exogenous ^13^C-labeled substrates, certain metabolic pathways can be studied using in vivo ^13^C MRS [36,37]. In our laboratory, several methods including an inverse detection method have been developed to measure different enzymatic reactions and their rate constants in vivo using ^13^C MRS [38,39,40,41,42,43]. 

Magnetization transfer can be incorporated into ^13^C MRS and the rate of CA-catalyzed carbon dioxide–bicarbonate exchange reaction can be measured quantitatively. Literature studies have suggested that CA inhibitors (CAIs) exert therapeutic effects on various neurodegenerative and psychiatric disorders [20,59,60,61,62,63,64,65,66,67]. Effect of CAIs and CA activators on carbon dioxide–bicarbonate saturation transfer can be monitored using in vivo MRS because they alter the rate of carbon dioxide–bicarbonate interconversion. 

### 2.1. Theory of ^13^C Magnetization Transfer Catalyzed by Carbonic Anhydrase

Magnetization transfer spectroscopy can measure fast enzymatic reactions [68,69,70]. The concentration of dissolved free carbon dioxide gas in brain tissue is approximately 1 mM at normal physiological conditions [71]. In contrast, bicarbonate concentration in the brain under normal physiological conditions is much higher (> 20 mM) [72] than CO_2_. Here we will provide a theoretical analysis of saturation transfer between carbon dioxide and bicarbonate catalyzed by CA using a two-site kinetic model that consists of a small CO_2_ pool and a large bicarbonate pool and quantitatively examine the effect of rapidly turning over CO_2_, which may require the use of relatively high radio frequency power for irradiation. The large difference between the CO_2_ and bicarbonate pool sizes also allows a quasi-steady state approximation of the dynamic longitudinal relaxation process of bicarbonate in the presence of its rapid exchange with the much smaller CO_2_ pool.

**Figure 1:**
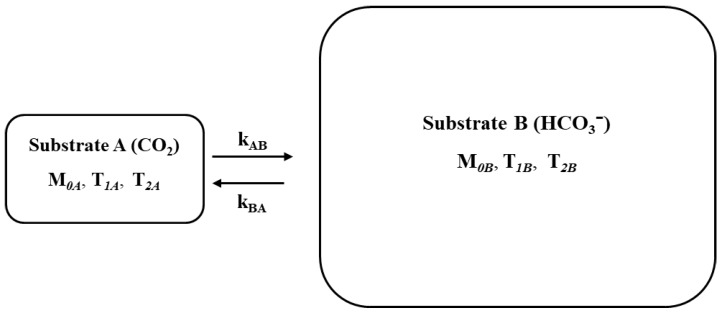
The two-site exchange diagram for CO_2_ ↔ HCO_3_^−^ catalyzed by carbonic anhydrase. *M*_0*A*_ and *M*_0*B*_ denote the magnetization of CO_2_ and HCO_3_^−^ at thermal equilibrium. *T*_1*A*_, *T*_1*B*_ and *T*_2*A*_, *T*_2*B*_ are their respective longitudinal and transverse relaxation times without any chemical exchange. *k_AB_* and *k_BA_* represent the pseudo-first-order rate constants of the unidirectional CO_2_ → HCO_3_^−^ hydration and HCO_3_^−^ → CO_2_ dehydration reactions, respectively.

The rapid interconversion between the small carbon dioxide pool (A) resonating at 125.0 ppm and the large bicarbonate pool (B) resonating at 160.7 ppm (Figure 1) can be quantitatively described following the analysis of the α-ketoglutarate-glutamate exchange system [36]. The irradiating radio frequency pulse is applied along the *x*-axis in the radio frequency rotating frame centered at the resonant frequency of the CO_2_^13^C spin at 125.0 ppm. The amplitude of this irradiating radio frequency pulse is designated as ω_1_. The magnitude of the *x*, *y*, *z* magnetizations of the ^13^C spin of CO_2_ (*M_xA_*, *M_yA_*, *M_zA_*) and those of bicarbonate (*M_xB_*, *M_yB_*, *M_zB_*) are governed by the Bloch-McConnell equations [73,74] for CA-catalyzed rapid interconversion between CO_2_ and bicarbonate:

(1) $$\frac{dM_{xA}}{dt}=-\frac{M_{xA}}{T_{2A}}-k_{AB}M_{xA}+k_{BA}M_{xB}$$

(2) $$\frac{dM_{yA}}{dt}=\omega_{1}M_{zA}-\frac{M_{yA}}{T_{2A}}-k_{AB}M_{yA}+k_{BA}M_{yB}$$

(3) $$\frac{dM_{zA}}{dt}=-\omega_{1}M_{yA}-\frac{M_{zA}-M_{0A}}{T_{1A}}-k_{AB}M_{zA}+k_{BA}M_{zB}$$

(4) $$\frac{dM_{xB}}{dt}=-\Delta \omega M_{yB}-\frac{M_{xB}}{T_{2B}}+k_{AB}M_{xA}-k_{BA}M_{xB}$$

(5) $$\frac{dM_{yB}}{dt}=\Delta \omega M_{xB}+\omega_{1}M_{zB}-\frac{M_{yB}}{T_{2B}}+k_{AB}M_{yA}-k_{BA}M_{yB}$$

(6) $$\frac{dM_{zB}}{dt}=-\omega_{1}M_{yB}-\frac{M_{zB}-M_{0B}}{T_{1B}}+k_{AB}M_{zA}-k_{BA}M_{zB}$$

In the above equations, *Δω* denotes the chemical shift difference between the ^13^C spins of bicarbonate and CO_2_; *T*_1*B*_, *T*_1*A*_, *T*_2*B*_, and *T*_2*A*_ are *T*_1_ and transverse relaxation times (*T*_2_); *k_BA_* and *k_AB_* are the pseudo-first-order rate constants of the unidirectional dehydration reaction bicarbonate → CO_2_, and hydration reaction CO_2_ → bicarbonate, respectively. 

Because the concentration of CO_2_ is much smaller than that of bicarbonate the standard quasi- steady-state assumption [74] in kinetics analysis is applicable here:

(7) $$\frac{dM_{xA}}{dt}\approx \frac{dM_{yA}}{dt}\approx \frac{dM_{zA}}{dt}\approx 0$$

At equilibrium, we have

(8) $$k_{BA}M_{0B}=k_{AB}M_{0A}$$

where *M*_0*A*_ and *M*_0*B*_ represent the thermal equilibrium magnetizations of the ^13^C spins of CO_2_ and bicarbonate, respectively. 

When CO_2_ is saturated by a radio frequency pulse that does not act on the bicarbonate signal directly, we observe a change in the steady state magnetization of bicarbonate *ΔM_zB_*. The expression for *k_BA_* can be shown to be the same as that for glutamate → α-ketoglutarate reaction given in ref. [36] despite that the concentration of CO_2_ is orders of magnitude higher than the concentration of α-ketoglutarate:

(9) $$k_{BA}=\frac{\left(1+\frac{pq}{\omega_{1}^{2}}\right)\frac{\Delta M_{zB}}{M_{0B}}}{T_{1B}^{sat}}$$

where $\Delta M_{zB}\equiv M_{0B}-M_{zB}^{ss}$, T1Bsat≡T1B(1+kBAT1B), $p\equiv \frac{1}{T_{2A}}+k_{AB}-\frac{k_{AB}k_{BA}T_{2B}}{1+k_{BA}T_{2B}}$, and $q\equiv \frac{1}{T_{1A}}+k_{AB}-\frac{k_{AB}k_{BA}T_{1B}}{1+k_{BA}T_{1B}}$ according to the expanded Bloch-McConnell equations (Equations (1)–(6)). 

Significant errors in measuring *k_BA_* may occur when the longitudinal magnetization of the ^13^C spin of bicarbonate at 160.7 ppm is significantly perturbed by the irradiating field *ω*_1_ [75,76] placed at 125.0 ppm. Using *M*_0*A*_ = 1 mM, *M*_0*B*_ = 20 mM, *k_BA_* = 0.28 s^−1^ and *T*_1*B*_ = 9.6 s [43], *p* and *q* can be estimated by assuming *k_BA_T*_2*B*_ << 1 which can be justified based on the relatively narrow in vivo bicarbonate linewidth. Using Equation (8) we obtain *k_AB_* and therefore *p* ≈ 5.6 s^−1^ and *q* ≈ 1.5 s^−1^ and *pq* ≈ 8.4 s^−2^. For < 1% error in *k_BA_* originated from Equation (9) the theoretically minimum *ω*_1_ is calculated to be merely ~5 Hz. As a nominal *ω*_1_ of 50 Hz was used experimentally to saturation CO_2_ no significant error is expected from incomplete saturation of CO_2_. 

Because *ω*_1_ is sufficiently large *k_BA_* as a function of ω_1_ ($⟫\sqrt{pq}$) and *Δω* can be derived from the full Bloch-McConnell Equations (1)–(6) for the bicarbonate steady-state magnetization. Again, this expression (Equation (10)) is found to assume the same form as that of the α-ketoglutarate ↔ glutamate exchange system [36] in spite of the large differences between the two exchange systems including the large difference in chemical shift separation between *A* and *B*:

(10) $$k_{BA}=\frac{\Delta M_{zB}}{M_{0B}}\left(\frac{1}{T_{1B}^{sat}}+r\right)-r$$

where $r\equiv \frac{\omega_{1}^{2}T_{2B}^{sat}}{1+\Delta \omega^{2}T_{2B}^{sat2}}$, $T_{2B}^{sat}\equiv \frac{T_{2B}}{1+k_{BA}T_{2B}}$. When $\omega_{1}⟫\sqrt{pq}$ complete saturation of CO_2_ is achieved. When the separation between the resonance signals of CO_2_ and bicarbonate is sufficiently large, i.e., Δω⟫ω1T1BsatT2Bsat, r in Equation (10) becomes negligible. At 7 Tesla the chemical shift difference between bicarbonate and CO_2_ *Δω* is 3562 Hz. From Equation (10) and because $\Delta \omega T_{2B}^{sat\;}$>> 1, *r* ≈ 0.002–0.004 s^−1^ for *ω*_1_ = 50 Hz and *T*_2*B*_ ≈ 0.05–0.1 s. Therefore, any error in *k_BA_* due to RF spill over is negligible, thanks to the large chemical shift dispersion at the high magnetic field strength of 7 Tesla. At lower field strength such as 1.5 Tesla, RF spill over can still be made negligible because of the very low *ω*_1_ threshold required for complete CO_2_ saturation. Therefore, with proper experimental design, both Equations (9) and (10) reduce to the well-known classical formula for saturation transfer [68,69,77]:

(11) $$k_{BA}=\frac{\frac{\Delta M_{zB}}{M_{0B}}}{T_{1B}^{sat}}$$

or

(12) $$k_{BA}=\frac{\frac{\Delta M_{zB}}{M_{zB}^{ss}}}{T_{1B}}$$

From the above analysis, Equations (11) and (12) [68,69,77] are valid for extracting *k_BA_* of bicarbonate-CO_2_ exchange accurately from data acquired in a steady-state magnetization (saturation) transfer experiment under the conditions of $pq⟪\omega_{1}⟪\Delta \omega \sqrt{T_{1B}^{sat}T_{2B}^{sat}}$. These conditions can be readily met using modern scanners because of the relatively large chemical shift separation between carbon dioxide (125.0 ppm) and bicarbonate (160.7 ppm). Equation (12) becomes Equation (1) in ref. [43] when the recycle delay is infinitely long. The above analysis therefore validated the simplified treatment used in ref. [43] for extracting *k_BA_* from our in vivo measurement.

Because of its small pool size, the magnetization of CO_2_ is approximately in instantaneous equilibrium with the large bicarbonate pool. Under conditions of complete radio frequency saturation of CO_2_ and no radio frequency perturbation of bicarbonate Equation (6) describes a longitudinal relaxation process for bicarbonate with a single time constant. When CO_2_ is not saturated, the dynamics of bicarbonate longitudinal relaxation is described by the analytical solutions to the classic Bloch-McConnell equations for two-site exchange [78]. The longitudinal relaxation behavior of bicarbonate with radio frequency saturation of CO_2_ is approximately the same as that in the absence of any exchange with CO_2_.

### 2.2. ^13^C Magnetization Transfer MRS

The ^13^C magnetization (saturation) transfer technique used to measure the bicarbonate dehydration rate constant in human brain [43] is summarized here. Although the original MRS method employed surface coil for spatial localization we emphasize that the more precise gradient-based localization techniques can also be used, thanks to the large in vivo magnetization transfer effects catalyzed by carbonic anhydrase.

#### 2.2.1. Magnetic Resonance Hardware

A two-channel spectrometer is required for measuring carbonic anhydrase using in vivo ^13^C saturation transfer experiments. Our in vivo ^13^C MRS magnetization transfer experiments for measuring carbonic anhydrase in the human brain [43] were performed on a Siemens Magnetom 7 Tesla scanner (Siemens Healthcare, Erlangen, Germany). A home-made RF coil assembly consisted of a circular ^13^C coil with a diameter of 7 cm and a quadrature half-volume proton coil which were mounted on three half-cylindrical plastic tubes, respectively. No proton blocking L-C tank circuit was found to be necessary for the ^13^C coil. A slotted RF shield made of copper foil with equally spaced gaps was placed on the outer surface of the lower plastic tube. The space between adjacent gaps was approximately 5 cm with one 1000 pF capacitor used to bridge each gap. Each proton loop had a single-tuned ^1^H cable trap (RG-316). A ^13^C/^1^H dual-tuned cable trap was placed inside an RF-shielded box and connected to the ^13^C coil. At proton frequency (300 MHz), RF isolation between the two proton loops was −20 dB. The RF isolation between the ^13^C coil and the two proton loops were −40 dB. At ^13^C frequency (75 MHz), isolation between the ^13^C coil and the two proton coils was −38 dB. The home-made ^13^C/^1^H coil system was connected to the 7 Tesla scanner through a commercially available interface box provided by Quality ElectroDynamics (Mayfeld Village, OH, USA).

#### 2.2.2. ^13^C Magnetization Transfer MRS Pulse Sequence

**Figure 2:**
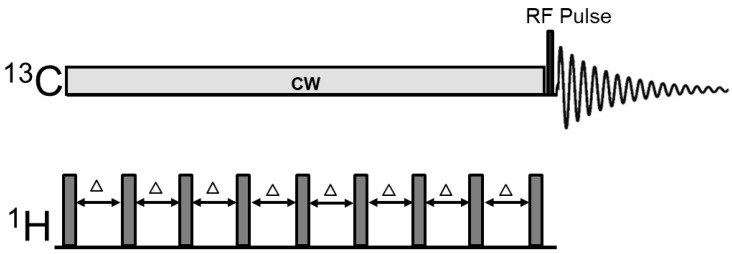
Radiofrequency pulse sequence for the ^13^C saturation transfer experiments. ^1^H→^13^C heteronuclear Nuclear Overhauser Enhancement (NOE) was generated by saturating proton signals using evenly spaced non-selective hard pulses. A continuous wave (CW) ^13^C pulse or a train of spectrally selective shaped ^13^C pulses was used for radiofrequency saturation at CO_2_ resonance or at the control frequency on the opposite side of bicarbonate. For excitation, a ^13^C block pulse was used. Δ: Delay between proton pulses (48 ms).

The RF pulse sequence for measuring carbonic anhydrase-catalyzed magnetization transfer is depicted in Figure 2. The ^13^C magnetization transfer effect catalyzed by carbonic anhydrase can be detected by spatial localization using either field gradient or surface coil with an interleaved acquisition scheme. Radio frequency saturation of CO_2_ was conducted by continuous wave (CW) or a train of evenly spaced spectrally selective shaped pulses for acquiring saturation transfer spectra using the ^13^C channel. To acquire the control spectra, the identical continuous wave saturating pulse or spectrally selective shaped pulses were placed at an equal spectral distance from the observed ^13^C spin of bicarbonate but on the opposite site of the CO_2_ resonance. The following interleaved acquisition scheme was used: {control irradiation–bicarbonate excitation–acquisition}–{carbon dioxide saturation–bicarbonate excitation–acquisition} to minimize the effect of changes in the signal intensity of ^13^C-labeled bicarbonate during MRS scan. For our 7 Tesla study [43], the excitation hard pulse (250 μs) was placed on-resonance (at 160.7 ppm, the resonance frequency of bicarbonate). A 50 ms composite pulse block was repeatedly applied from the end of data acquisition to the start of excitation by the ^13^C hard pulse. Each composite pulse block consists of a 1.0 ms proton hard pulse for generating broadband heteronuclear nuclear Overhauser enhancement and a 48.0 ms continuous wave ^13^C pulse (nominal γB_1_ = 50 Hz) for saturating carbon dioxide at 125.0 ppm or for irradiation at the control frequency. Proton decoupling was not conducted because the proton of bicarbonate is in very rapid exchange with tissue water and it is self-decoupled from ^13^C spins via its chemical exchange with water. Each pair of spectra for measuring saturation transfer signal difference consisted of 24 free induction decays (number of averages = 24 with 12 averages for each irradiated frequency). The following acquisition parameters were used: spectral width = 8 kHz, data points = 2048, acquisition time = 256 ms, and recycle delay = 30 s.

For absolute quantification of the bicarbonate dehydration rate constant, the longitudinal relaxation time of the observed ^13^C spin of bicarbonate was measured by a $T_{1B}^{sat}$ or $T_{1B}$ null experiment ($\exp\left(\frac{T_{1null}}{T_{1B}^{sat}}\right)+\exp\left(-\frac{TR-T_{1null}}{T_{1B}^{sat}}\right)=2$). *TR* is the repetition time. *T*_1*null*_ is the time when the ^13^C spin of bicarbonate magnetization reaches zero. For *TR* >> *T*_1*B*_, $\exp\left(\frac{T_{1null}}{T_{1B}^{sat}}\right)=2$. The *T_1null_* of bicarbonate ($T_{1B}$) with optional saturation of CO_2_ ($T_{1B}^{sat}$) was measured using a 30-ms hyperbolic secant inversion pulse for adiabatic inversion with a much longer recycle delay of 55 s followed by direct excitation and detection of free induction decay of ^13^C-labeled bicarbonate spins. 

### 2.3. Isotope Labeling Strategies and ^13^C MRS of the Carboxylic/Amide Spectral Region

Natural abundance of ^13^C is only 1.1%, so exogenous ^13^C -labeled glucose was administered to human subjects to introduce ^13^C labels to CO_2_ and bicarbonate molecules. For in vivo determination of carbonic anhydrase-catalyzed interconversion between CO_2_ and bicarbonate uniformly ^13^C labeled glucose is an excellent choice as all six ^13^C labels on a glucose molecule are eventually passed to CO_2_ and bicarbonate via the pyruvate dehydrogenase reaction and the tricarboxylic acid cycle. Use of uniformly ^13^C labeled glucose leads to maximum ^13^C enrichment of CO_2_ and bicarbonate. 

**Figure 3:**
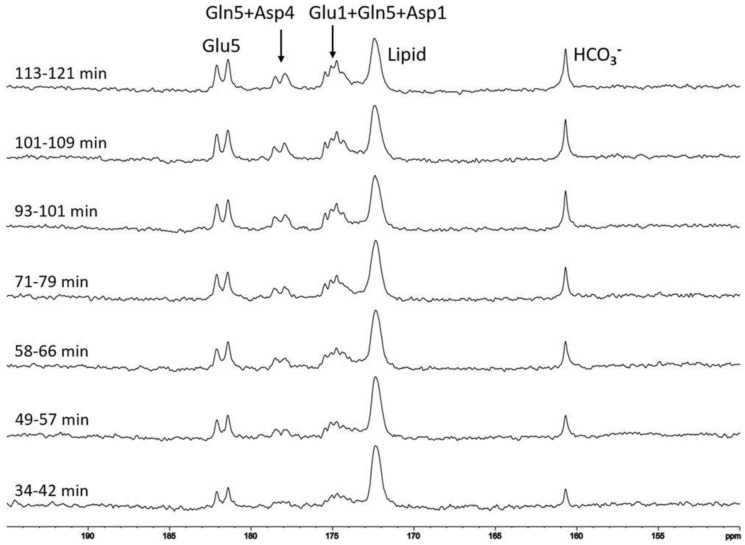
A typical time-course of control spectra acquired from a single subject after oral administration of [U-^13^C_6_] glucose without proton decoupling. Each spectrum was acquired with recycle delay = 30 s, spectral width = 8 kHz, number of data points = 2048, number of averages = 12, and line broadening = 8 Hz. The time interval indicates the beginning and end of acquisition following oral glucose intake. Lipid: carboxylic carbons of natural abundance lipids (172.5 ppm), Glu5: glutamate C5 (182.0 ppm), Glu1: glutamate C1 (175.4 ppm), Gln5: glutamine C5 (178.5 ppm), Gln1: glutamine C1 (174.8 ppm), Asp4: aspartate C4 (178.3 ppm), Asp1: aspartate C1 (175.0 ppm) (reprinted from ref. [43]. https://creativecommons.org/licenses/by/4.0/).

**Figure 4:**
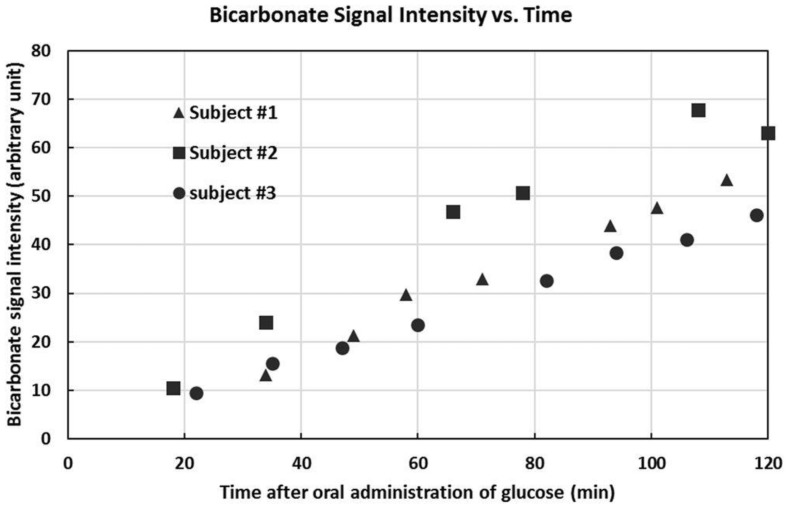
Bicarbonate signal intensities as a function of time after oral administration of [U-^13^C_6_] glucose. The glucose level was different in different subjects during the scan time. Bicarbonate signal increased monotonically (reprinted from ref. [43]. https://creativecommons.org/licenses/by/4.0/).

Since the ^13^C labeling kinetics of the tricarboxylic acid cycle is not of concern for measuring the carbonic anhydrase reaction, ^13^C labeled glucose can be conveniently administered orally. We administered a solution of 20% *w/w* 99% enriched [U-^13^C6] glucose at a dose of 0.75 g [U-^13^C_6_] glucose per kg of body weight before initiation of ^13^C MRS scans. All subjects underwent at least 12-h fasting before the MRS study. Following oral administration of glucose, ^13^C labels are rapidly incorporated into glutamate, glutamine, aspartate, and bicarbonate molecules. In the carboxylic/amide spectral region, a steady increase in the signal intensity of glutamate (C5 and C1), glutamine (C5 and C1), aspartate (C4 and C1), and bicarbonate were observed (see Figure 3 and Figure 4).

Variations in ^13^C signal intensity of bicarbonate may cause errors in measuring the saturation transfer effect which requires subtraction of two spectra acquired 30 s apart. As shown in Figure 3 and Figure 4, variation in ^13^C signal intensity of bicarbonate is much slower on a time scale measured by hours. Therefore, changes in the intensity of ^13^C-labeled bicarbonate is negligible over a period of 30 s, which is the recycle delay of our interleaved acquisition scheme shown in Figure 2. The slow ^13^C labeling kinetics of bicarbonate following oral intake of ^13^C-labeled glucose can be attributed to the damping effect exerted by the stomach and to the large size of label trapping pools such as cerebral glutamate. For measuring the in vivo activity of carbonic anhydrase, the absolute ^13^C fractional enrichment of bicarbonate is not of concern except that higher ^13^C fractional enrichment leads to higher SNR as Equations (11) and (12) narrates that only the relative change in the ^13^C-labeled bicarbonate signal is needed to calculate the bicarbonate dehydration rate constant. Therefore, from a technical point of view, the optimal time to measure carbonic anhydrase activity following administration of exogenous ^13^C labels is when the intensity of ^13^C-labeled bicarbonate reaches maximum. Furthermore, because only the relative change in ^13^C-labeled bicarbonate signal intensity upon saturating CO_2_ is used to calculate k_BA_ so differences in individual subject’s response to glucose administration will not affect the accuracy of carbonic anhydrase activity measurement.

The signal of ^13^C-labeled carbon dioxide has not been observed in the human brain. This could be due to its small pool size (~1 mM), the off-resonance effect of the excitation pulse, the presumably very long T_1_ of the unprotonated CO_2_, and possible line-broadening of the electrically neutral CO_2_ molecule in vivo. In Figure 2, a 250 us block pulse was used to excite the bicarbonate signal. Because the signal of CO_2_ was observed in early in vitro studies of protein and membrane systems [79], significant CO_2_ line-broadening in vivo is highly unlikely. Because tissue pH and pCO_2_ were not measured, the total concentration of bicarbonate cannot be determined. Fortunately, because the pseudo first-order dehydration rate constant is derived from Equations (9)–(12), the absolute concentration of CO_2_ and bicarbonate have no effect on the absolute quantification of the bicarbonate dehydration reaction rate constant, if sufficient signal-to-noise ratio is achieved. 

### 2.4. Characterization of Carbonic Anhydrase Reaction in the Human Brain

**Figure 5:**
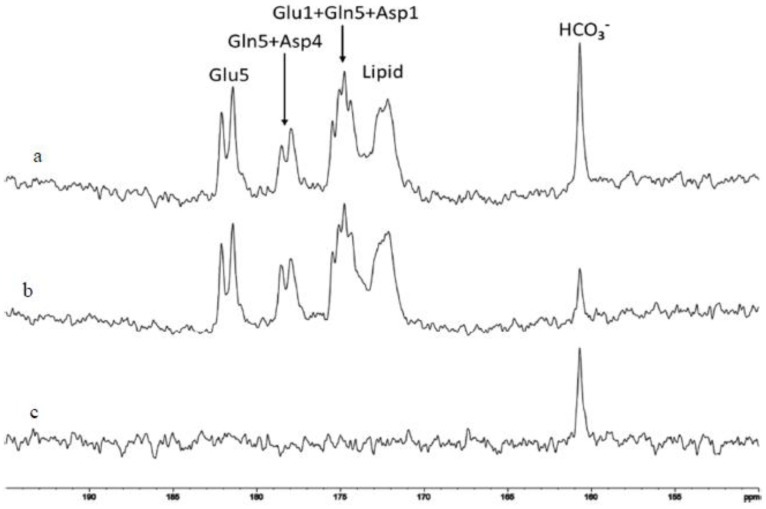
^13^C saturation transfer effect catalyzed by carbonic anhydrase (CA) in the human brain. Spectra were measured from a single subject between 118 and 130 minutes after oral administration of 20% [U-^13^C_6_] glucose. (**a**) control spectrum with ^13^C irradiation at 228 ppm; (**b**) with saturation of carbon dioxide at 125.0 ppm; (**c**) difference spectrum.

For the two-site exchange reaction depicted in Figure 1, RF saturation of CO_2_ is carried over to bicarbonate due to the interconversion between the two. Therefore, RF saturation of CO_2_ causes a reduction in the magnetization of bicarbonate. This reduction in the magnetization of bicarbonate perturbs its thermal equilibrium, triggering its longitudinal relaxation toward regaining its thermal equilibrium. These two opposing forces reach a steady state and result in an attenuated bicarbonate magnetization. Figure 5 shows the spectra of ^13^C saturation transfer results measured between 118 and 130 min after the oral administration of [U-^13^C_6_] glucose. The top spectrum (Figure 5a) is the control spectrum. Such control irradiation is unnecessary at high magnetic field as our previous studies had shown that there were no detectable nonspecific ^13^C off-resonance magnetization transfer effects [37,38] and Section 2.1 indicates that RF spillover effect is negligible at 7 Tesla. In Figure 5a the full signal intensity of ^13^C-labeled bicarbonate was recorded. The middle spectrum (Figure 5b) was acquired with RF saturation of carbon dioxide at 125.0 ppm. A large reduction in the bicarbonate signal intensity is seen due to carryover of saturation from the CO_2_ magnetization to bicarbonate. The bottom spectrum (Figure 5c) is the difference spectrum obtained by subtraction of Figure 5b from Figure 5a. A large reduction of the signal intensity of bicarbonate by 72% ± 0.03 (*n* = 3) due to carbon dioxide saturation transfer was measured for the first time in the human brain [43]. This represents the largest known saturation transfer effect among chemicals observable in vivo in human subjects. The bicarbonate dehydration rate constant (*k_BA_*) in the human brain was found to be 0.27 ± 0.03 sec^−1^ (*n* = 3).

## 3. Future Applications of in Vivo MRS of Carbonic Anhydrase Reaction

The non-invasive in vivo MRS technique for measuring the carbonic anhydrase reaction has the exciting potential to characterize carbonic anhydrase activities in many biomedical applications, especially in studying brain disorders where in most situations biopsy is not feasible. Here we provide a brief literature survey of the roles of carbonic anhydrase and its inhibitors in basic neuroscience and in many neurological and psychiatric diseases. Potential applications of in vivo MRS of carbonic anhydrase in these studies will be discussed. 

Epilepsy is a complex neurological disorder of varying etiology manifested by abnormal excessive or synchronous neuronal activity in the brain. An epileptic episode is linked to fast alterations in the neuron ionic compositions [35,80,81,82,83,84,85,86,87]. In vivo MRS has been applied to studying epilepsy and its treatment for decades [88]. Most MRS studies have focused on measuring N-acetyl aspartate as a neuronal marker, glutamate as a marker of the excitatory glutamatergic neurons, as well as GABA and GABAergic system [89] in epilepsy patients. Of them, detection of a deficit in GABA level and the elevation of GABA level and corresponding reduction in seizure activities following treatment using vigabatrin have been a major milestone in the technical development and clinical application of in vivo MRS [90].

Carbonic anhydrase inhibitors (CAIs) are known to exhibit anticonvulsant properties. Some carbonic anhydrase inhibitors are clinically used to treat epilepsy. In the CNS carbonic anhydrase inhibition enhances inhibitory neurotransmission [91]. Augmentation of inhibition following carbonic anhydrase inhibition has been well studied at the level of voltage- and ligand-gated ion channels and gap junctions [92]. Correlation between extent of carbonic anhydrase inhibition and GABAergic effect of carbonic anhydrase inhibition is well established, so in vivo MRS is well-positioned to study GABA-carbonic anhydrase interactions in patients with neurological or psychiatric diseases.

Historically, CAI acetazolamide was marketed as a diuretic drug and concurrently its anticonvulsant property was discovered [93]. In addition, use of CAI in the treatment of several psychiatric disorders has also been reported. Analysis of protein–protein interaction network of schizophrenia associated genes and drug–protein interactome resulted in 12 potential repurposable drugs including acetazolamide [94]. Many genes within this network showed association with various neuropsychiatric disorders and a few of these genes were acetazolamide targets [94]. Additionally, recent proteomic studies of brain disorders such as schizophrenia and major depression have also revealed marked alterations in CA expression [44,95].

**Figure 6:**
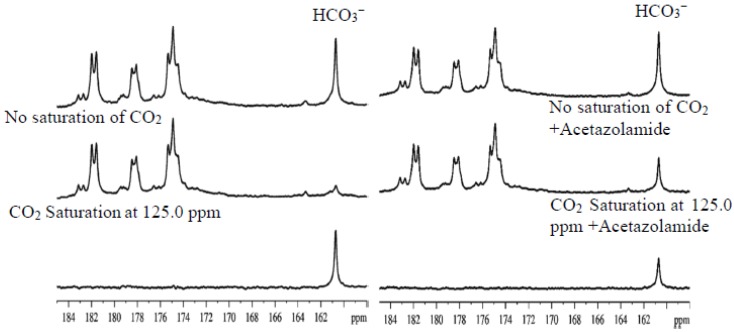
In vivo ^13^C magnetization transfer effect of carbon dioxide–bicarbonate exchange in rat brain before (**left**) and after (**right**) carbonic anhydrase inhibition. Upper traces: no saturation of carbon dioxide. Middle traces: with saturation of carbon dioxide at 125.0 ppm. Lower traces: difference spectra. The ^13^C magnetization transfer effect of the carbon dioxide–bicarbonate exchange was significantly reduced after blockade of carbonic anhydrase (adapted from ref [40] with permission from John Wiley and Sons Ltd.).

**Figure 7:**
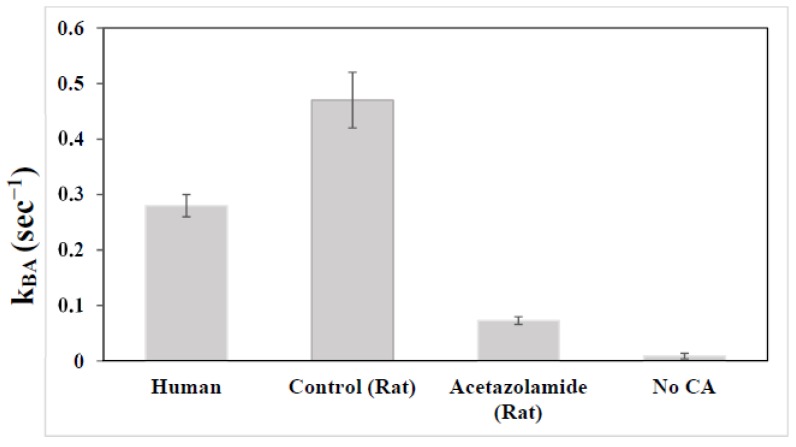
Pseudo first-order unidirectional bicarbonate dehydration rate constant *k_BA_* determined from healthy human subjects, control rats, rats treated with acetazolamide, and a phantom (standard deviation is plotted as error bars).

Several clinically used antipsychotic drugs have been screened against CA and many of them inhibit CA at micromolar concentration [96]. Interestingly, the well-known selective serotonin reuptake inhibitors fuoxetine, sertraline, and citalopram are strong CA activators [97]. In a previous double-blind crossover randomized placebo-controlled clinical trial, adjunctive acetazolamide was found to significantly improve both positive and negative symptoms in treatment-refractory schizophrenia patients [98,99]. The beneficial effects of CA inhibitor acetazolamide in the treatment of schizophrenia and bipolar disorder have also been reported by other studies [54,55,93,94,100]. In a previous animal study [40], we showed that administration of acetazolamide to rodents led to a significant reduction in the rate of bicarbonate dehydration in vivo. This reduction in carbonic anhydrase activity caused by acetazolamide is reflected by a markedly reduced ^13^C magnetization transfer effect readily quantifiable by in vivo magnetization transfer MRS [40] (Figure 6 and Figure 7). Figure 7 also compares the bicarbonate dehydration rate constants measured from human and rodent brains. Interestingly, the carbonic anhydrase activity in healthy human subjects as measured by the bicarbonate dehydration rate constant is notably lower than in control rats not treated with carbonic anhydrase inhibitor acetazolamide.

## 4. Conclusions

A growing body of evidence links altered carbonic anhydrase expression with many diseases including major neurological and psychiatric disorders. Carbonic anhydrase inhibitors have been in clinical use for decades with a wide range of therapeutic effects. However, there had been no in vivo techniques to directly and noninvasively assess carbonic anhydrase activity and to monitor treatments that target carbonic anhydrase activity until the recent emergence of in vivo ^13^C magnetization transfer spectroscopy directly measuring the carbonic anhydrase activity. In vivo MRS of carbonic anhydrase reaction has great importance for studying the role of carbonic anhydrase in brain disorders and to evaluate target engagement of carbonic anhydrase inhibitors and activators in the human brain. It is expected that in vivo MRS will play an important role in our understanding of carbonic anhydrase and its modulation in many diseases and their treatments. 

## Abbreviations

CA	Carbonic anhydrase
CAI	Carbonic anhydrases inhibitor
MRS	Magnetic resonance spectroscopy
